# Practices and views of audiologists regarding aural rehabilitation services for adults with acquired hearing loss

**DOI:** 10.4102/sajcd.v63i1.155

**Published:** 2016-09-29

**Authors:** Musa Makhoba, Neethie Joseph

**Affiliations:** 1 Discipline of Audiology, University of KwaZulu-Natal, South Africa

## Abstract

**Background:**

Hearing loss in adults is one of the leading disabilities globally. It is managed through aural rehabilitation for which there is a paucity of literature in South Africa. This raises the question of interest, the integrity of holistic service provision amongst audiologists and whether interest and challenges affect current practices.

**Objectives:**

To describe audiologists’ practices and views on aural rehabilitation services for adults, including interest and challenges experienced.

**Method:**

A descriptive online survey was completed by 45 of 1440 invited practicing audiologists who were members of the two national professional associations in South Africa. Each association emailed the questionnaire link to all its members. Data were analysed using the Statistical Package for the Social Sciences version 21, and included the paired samples *t*-test and chi-squared tests.

**Results:**

The most provided services were hearing aids (81.4%), communication strategies training (69.8%) and informational counselling (79.8%). A strong interest was reported by most for each service. Challenges included limited client compliance, unaffordability of services, limited undergraduate training, language barriers, unrealistic expectations and individual differences. Statistically significant differences between service provision, interest and challenges indicated that these are influential but not individually significant to service provision.

**Conclusion:**

Imbalanced service provision, high interest and many more challenges are experienced. These factors contribute but are not solely markedly influential in service provision.

## Introduction

### Background

Hearing loss in adults is one of the most common disabilities globally (World Health Organization [WHO], 2011), especially in countries in sub-Saharan Africa (Adoga, Nimkur & Silas, 2010). It was reportedly the third most prevalent disability in 2007 in South Africa (Statistic South Africa [Stats SA], 2007). Adult South Africans are exposed to many possible causes of hearing loss such as excessive noise, ototoxic medication and diseases such as HIV-AIDs that lead to hair cell damage (Khoza & Ross, 2002). These adults are at risk of experiencing hearing-related communication difficulties such as poor speech discrimination and reduced access to environmental sounds (Heine & Browning, 2002), emotional withdrawal (Pronk *et al*., 2011) and poor quality of life (Joore, Potjewijd, Timmerman & Anteunis, 2002). Such difficulties may be effectively managed through aural rehabilitation provided by audiologists (Tye-Murray, 2009). Aural rehabilitation may help reduce participation limitation and facilitate better personal and environmental strategies to reduce the disabling effects of hearing loss (WHO, 2001).

**FIGURE 1:**
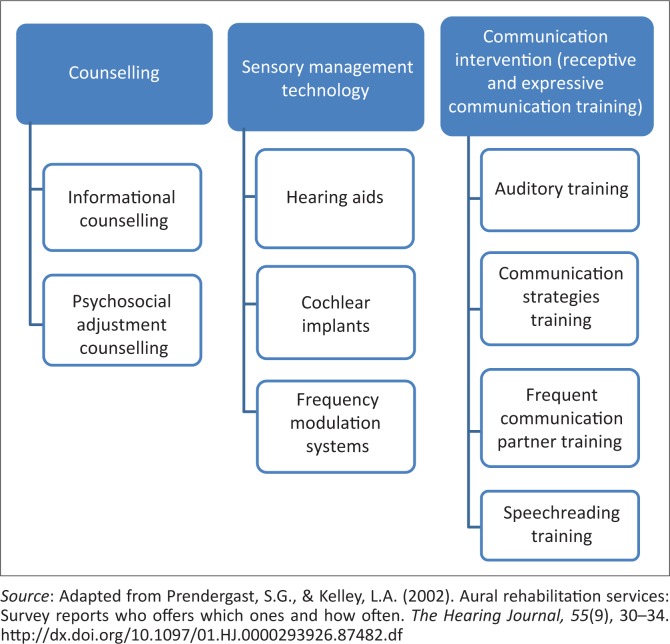
Aural rehabilitation components and services.

Aural rehabilitation is defined as ‘any device, procedure, information, interaction, or therapy which lessens the communicative and psychosocial consequences of a hearing loss’ (Ross, cited in Bally & Bakke, 2007, p. 125). In accordance with this definition, the current study adapted a list of services from the study conducted by Prendergast and Kelly (2002), who investigated the provision of various aspects of aural rehabilitation services by audiologists. These services were organised into three main components of a comprehensive aural rehabilitation programme, including sensory management, communication training and counselling (Figure 1) (Boothroyd, 2007). However, interest in these services for adult clients has been reported to be on a decline since the 1970s (Schow, Balsara, Smedley & Whitcomb, 1993). Limited and imbalanced aural rehabilitation service provision and limited locally relevant literature in South Africa seems to be a concern to the profession of audiology as there is more focus on life-threatening conditions, which is typical in developing countries (Olusanya, 2004). Intervention with hearing aids seems to be the most preferred service by most audiologists, who tend to focus less on comprehensive aural rehabilitation provision (Naidoo, 2006; Sweetow & Palmer, 2005). Reasons for this trend are unknown, but it is likely owing to the fact that hearing aids are typically the starting point to enhance hearing prior to providing further intervention (Sweetow & Sabes, 2005).

Sensory management includes personal devices that enhance access to auditory stimulation. Communication training involves helping one learn to effectively communicate after acquiring a hearing loss. Counselling involves providing information while supporting the client psychologically and emotionally to effectively deal with changes experienced because of changed hearing.

### Aural rehabilitation counselling

The relationship between communication limitations and psychological or emotional well-being in adults with acquired hearing loss necessitates psychosocial adjustment counselling to optimise the client’s emotional well-being and therapeutic progress (Ciorba, Bianchini, Pelucchi & Pastore, 2012; Pronk *et al*., 2011).

Informational counselling entails sharing information that would improve the client’s knowledge about the rehabilitative process and its importance (Hartley, 2005; Kochkin, 2009; Schneider *et al*., 2010 as cited in Meyer & Hickson, 2012). This counselling includes information about hearing, the effects of hearing loss and the importance of intervention (Tye-Murray, 2009). It also helps the client formulate realistic expectations of the intervention (Tye-Murray, 2009). Informational counselling may be used to address issues of cultural and stereotypical beliefs that could lead to limited compliance with the aural rehabilitation programme, which is very important in South Africa where there is strong cultural diversity (De Andrade & Ross, 2005; Mackenzie, 1999). Thus, audiologists in South Africa should be trained to provide culturally and contextually relevant aural rehabilitation, including counselling (National Department of Health, 2012).

### Sensory management

Sensory management specifically through hearing aids is usually a first step in the intervention process. Cochlear implants on the contrary are less widely fitted, more costly, need extra training for audiologists and involve other medical professionals, making them less feasible in South Africa. Frequency modulation (FM) systems are also an effective treatment of peripheral hearing and processing difficulties (Sykes, 2010). However, there is limited literature on its use in South Africa. It was thus imperative that the current study investigates the provision of each sensory management service and the possible reasons for the limited provision of some of these services.

### Communication intervention

Communication intervention helps improve communication skills, satisfaction, benefit and reduce dissatisfaction from using personal sensory management devices, leading to less retuned sensory devices from unsatisfied users (Hawkins, 2005; Pienaar, Stearn, & Swanepoel, 2010; Stecker, Bowman, Yund, Herron & Roup, 2006; Sweetow & Palmer, 2005). Knudsen, Oberg, Nielsen, Naylor and Kramer (2010) reported a non-use rate of up to 40% of hearing aids fitted in Australia, the United Kingdom and other countries outside Africa. Storm (2007, as cited in Sweetow & Sabes, 2010) reports a return rate of 17% of hearing aids for refund if fitting was without further intervention. It appears that audiologists do not provide sufficient communication intervention or mostly only offer specific services that are less time consuming within this category. This could possibly be because of lack of time and being understaffed, besides other challenges. Auditory training involves helping the client learn to effectively listen during conversation while communication strategies training involves training to improve expressive communication skills (Tye-Murray, 2009). Speechreading training involves training to effectively use audio-visual skills to improve communication (Bishop & Miller, 2011; Tye-Muray, 2009). However, research has constantly indicated limited provision of speechreading training in comparison to other services, even in South Africa over the years (Millington, 2000; Naidoo, 2006; Prendergast & Kelly, 2002; Schow *et al*., 1993).

Frequent communication partner training involves training communication partners to effectively communicate with the person who has a hearing loss (Laplante-L`evesque, Hickson & Worrall, 2010). Such training seems to be provided insufficiently in South Africa (Naidoo, 2006). Reasons behind this are unknown, but it could be because of limited training even though the scope of practice and undergraduate training should include family-orientated approaches to rehabilitation (National Department of Health, 2014).

### Aural rehabilitation technology

Use of computer-based interventions could optimise service provision in South Africa. For instance, computer-assisted aural rehabilitation programmes reportedly save time and improve the organisation of the data on each client (Sweetow & Sabes, 2007). However, there seems to be limited literature regarding use of such technology locally.

Four common programmes discussed by Sweetow and Sabes (2007) are recognised in the current study, namely the computer-assisted speech-perception testing and training at the sentence level (CASPERSent), the computer-assisted tracking simulation (CATS), the computer-assisted speech training (CAST) and the listening and communication enhancement (LACE) programme. Little is known about the use of these programmes in South Africa. Further, it also seems that practical training using these programmes is limited, if at all it takes place in local training institutions.

Tele-audiology is another technological development to make audiology services more accessible, especially in remote rural areas (Lawrence, 2012; Nemes, 2010; Swanepoel, 2012). However, it seems to remain unused by many audiologists in South Africa and there is limited literature on its use as a means to provide aural rehabilitation services. Hence, there is limited information on its effects on addressing some of the challenges experienced in providing aural rehabilitation (Sweetow & Sabes, 2007). This lack of use could be because of such technology being relatively new in South Africa. Thus, more could be known about it in future, including its possible use in the provision of aural rehabilitation.

### Challenges with aural rehabilitation services

Lack of resources to provide aural rehabilitation such as time, tools and audiology staff is a global challenge (Swanepoel *et al*., 2010). South Africa is no exception, and this may have contributed negatively to audiology service provision as many audiologists rather provide what they can and not what they should (Fagan & Jacobs, 2012; Swanepoel, 2006). Binzer (2002) mentioned the lack of adequate reimbursement for providing aural rehabilitation services as one of the reasons that many audiologists do not provide those services. Non-compliance of clients with therapy also affects the provision of aural rehabilitation services (Sweetow & Sabes, 2010). In South Africa, socio-economic factors may also impact on service delivery with issues such as unaffordability of transport costs, which could affect attendance to aural rehabilitation sessions for low-income clients. This may have implications for client compliance and benefit from intervention. Lack of audiologists’ interest in aural rehabilitation was another challenge reported in the study by Prendergast and Kelly (2002). Little is known about challenges of aural rehabilitation specific to the South African context owing to limited relevant literature. Thus, the current study would provide information about the current status of aural rehabilitation service provision, challenges, interest and related factors as reported by audiologists in current practice.

## Methodology

### Study design and context

This non-experimental, descriptive study was conducted using a self-administered online survey questionnaire developed by the researcher to best investigate audiologists’ views and practices of aural rehabilitation in South Africa.

### Aim and objectives

The study’s aim was to describe audiologists’ practice and views on the provision of aural rehabilitation services to adults with acquired hearing loss.

Objectives were:

- to describe aural rehabilitation services provided by audiologists to adults with acquired hearing loss;
- to describe interest in aural rehabilitation and challenges experienced by audiologists in providing aural rehabilitation services to adult clients.

### Participants

The link to the questionnaire was sent to a total of 1440 potential participants, of which 1080 were members of the South African Speech-Language-Hearing Association (SASLHA) and 360 were members of the South African Association of Audiology (SAAA). This was in keeping with previous studies that surveyed aural rehabilitation services provided by audiologists who were members of associations (Prendergast & Kelly, 2002; Schow *et al*., 1993; Whitcomb, 1982, as cited in Millington, 2000). All participants had to be registered with at least one of the two South African Associations and the Health Professionals Council of South Africa, and be currently working in South Africa as an audiologist and speech therapist and/or audiologist.

A total of 45 returned questionnaires were usable. Twenty-three participants (51.11%) were speech therapist and audiologists, and 22 (45.89%) were audiologists. Twenty-three (51.11%) of the participants worked in private practice, and 12 (26.67%) worked in public hospitals. Five (11.11%) participants worked in universities and five worked in other settings. Most participants had 6–10 years of work experience. The majority were 30 years or younger, and female.

### Materials

The questionnaire was developed using a google form from the Google Drive system (Google, 2013). It was chosen as it was freely accessible and user friendly for the researcher. It consisted of 28 items including short, open-ended, multiple-choice, Likert-scale contingent questions, and checkboxes. The questionnaire allowed for different levels of probing within the predominantly close-ended questions, as a large sample size was anticipated. The questionnaire comprised five sections relating to demographics, detailed aspects of service provision, interest and challenges, and training and technological advancements in aural rehabilitation.

## Ethical considerations

Ethical clearance (HSS/0027/013M) was obtained from the Humanities and Social Sciences Ethics Committee of the University of KwaZulu-Natal. Participants remained anonymous as their email addresses were known only to the professional associations who distributed the questionnaire. Participants received an information sheet explaining the nature of the study and their rights as participants, and provided consent before gaining access to the questionnaire.

### Reliability and validity

The questionnaire was piloted on five conveniently selected audiologists working at a university to test for any need for improvements (Leedy & Ormrod, 2013). The pilot questionnaires were initially distributed via post together with a short suggestion form for participants to suggest necessary improvements in the structure, understandability and length. Participants’ feedback from the pilot helped to ensure a good face and content validity. The high relevance of the construct to the participants ensured that the construct validity was not compromised. The use of a subjective questionnaire carries the risk of compromising reliability, thus the researcher relied on the participants being open and honest. However, there were responses through which internal consistency was checked amongst different questions probing the same service. The pilot study resulted in structural changes to the questionnaire and the use of an online questionnaire to further simplify the data collection process.

### Research procedure

SASLHA and SAAA were requested to email the link to the questionnaire to all their members. Participants submitted the completed questionnaires anonymously directly to the researcher’s email by clicking the submit button. Completed questionnaires were available in the google form for analysis. The professional associations were asked to send a reminder to all their members after a 2-week period. After 5 weeks from the date of questionnaire distribution, data were transferred to the Statistical Package for Social Sciences (version 22) for analysis.

### Data analysis

Descriptive and inferential statistics were used to analyse the data. The paired samples *t*-test was used to assess for significance of a difference between two services for one variable such as audiologists’ interest in hearing aids compared with auditory training.

The chi-squared (*X*^2^) test was used to compare variables of the same service for statistical significance of difference. For instance, a comparison between service provision, interest and challenges for hearing aids was made. The same was done for other services as well.

For both the chi-squared and the paired samples *t*-test, the statistical significance level (*p*-value) was set at *p* ≤ 0.05. Therefore, a *p*-value of more than 0.05 would be an indication that there is no significant difference between variables being compared. Thus, there would be a high chance of a relationship between the variables being compared. A statistician advised on the selection of statistical tests suitable for the number of responses and sample size to ensure good quality of data analysis.

## Results

### Description of participants

While the sample size was relatively small, which could be attributed to limited practice or interest in the topic of aural rehabilitation, results provided an insight into current practices and views. The small sample size and unequal participant numbers precluded analysis such as the difference in practice between public and private audiologists.

### Aural rehabilitation service provision

All services were predominantly provided through individual-based sessions. Of the 20 provided reasons, 18 (90%) were for individual-based sessions, including personal preference by them or their clients, lack of client compliance, poor feasibility, time, space and financial constraints. Reasons for two (10%) participants using group sessions included it being beneficial to family and friends of the client.

**FIGURE 2:**
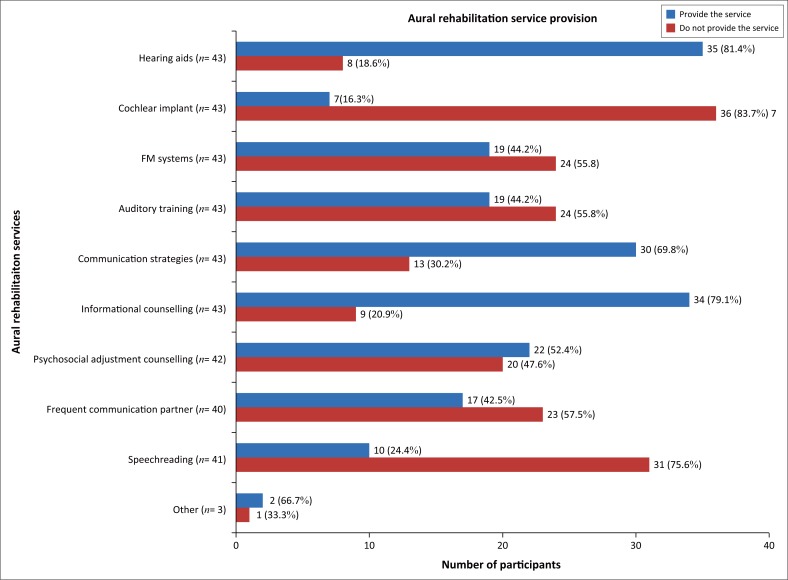
Specific aural rehabilitation services provided.

Hearing aids, communication strategies training and informational counselling were provided by more participants in comparison with other aural rehabilitation services (Figure 2).

Hearing aids were the most provided sensory management service by 81.4% of the 43 participants. Most (83.7%) of the 43 participants reported not providing cochlear implant intervention. The majority (79.1%) of the 43 participants reported providing informational counselling services which was significantly greater (*p* = 0.000) compared with the 52.5% that reported providing psychosocial adjustment counselling.

Significantly more (*p* = 0.001) participants provided communication strategies training compared with auditory training. There was a significant difference (*p* = 0.000) between communication strategies training and frequent communication partner training as more participants provided the former. Even more (75.6%) of the 41 participants did not provide speechreading training which was provided by significantly less participants (*p* = 0.033; *p* = 0.018) in comparison with frequent communication partner training and auditory training, respectively.

Five of the 11 participants provided reasons for not providing aural rehabilitation to adults. For instance, two (18.18%) only worked with paediatric clients, one (9.09%) only supervised other audiologists, one (9.09%) participant reported not being trained for cochlear implant mapping and one other (9.09%) participant reported language barriers, time and staff shortage.

Limited use of technology to aid aural rehabilitation service provision was reported. Use of computer-aided programmes seems limited in South Africa as none of the participants reported using the CATS, CASPERsent or CAST programmes. The LACE was used by eight (27.6%) of the 29 participants that responded to the question. Eleven (37.9%) participants used other programmes such as cochlear implant programmes, Earena, hearing aid programmes, internet-based resources and questionnaires such as the COSI. Five participants provided reasons for not using computer-aided programmes, including lack of resources, lack of awareness, and one participant reported using hearing aid software programmes instead, which she thought served the same purpose.

Four (13.3%) of the 30 participants that responded to the question on tele-audiology reported using it, amongst which one did not use it for aural rehabilitation and the others did not specify. Twenty-one participants provided reasons for not using tele-audiology. Six (28.57%) did not see a need; eight (38, 09%) reported lack of resources; and six (28.57%) reported lack of knowledge, skills or training on its use. One (4.76%) participant reported that she never thought tele-audiology could be used for aural rehabilitation services.

### Audiologists’ views on their interest in aural rehabilitation

Contrary to limited service provision, a strong interest was reported. For each aural rehabilitation service, the majority of the participants that did not already provide it reported being keen or very keen on providing it. Participants already providing aural rehabilitation were asked if they found each service interesting. Most found each service interesting or strongly interesting (Table 1).

**TABLE 1:** Interest in specific aural rehabilitation services provided.

AR service provided	Strongly not interesting		Not interesting		Neutral		A little interesting		Strongly interesting	
	*n*	%	*n*	%	*n*	%	*n*	%	*n*	%
Hearing aids (*n* = 35)	5	14.29	1	2.86	0	-	7	20	22	62.86
Cochlear implants (*n* = 5)	0	-	0	-	0	-	1	20	4	80
FM systems (*n* = 20)	2	10	0	-	0	-	10	50	8	40
Auditory training (*n* = 26)	2	7.69	1	3.85	3	11.54	7	26.92	13	56.52
Communication strategies training (*n* = 31)	3	9.68	1	3.23	3	9.68	9	29.03	15	48.39
Informational counselling (*n* = 35)	3	8.57	2	5.71	2	5.71	10	28.57	18	51.43
Psychosocial adjustment (*n* = 26)	2	7.69	0	-	3	11.54	6	23.08	15	57.69
Frequent communication partner training (*n* = 22)	1	4.55	1	4.55	2	9.09	7	31.82	11	50
Speechreading (*n* = 17)	1	5.88	4	23.53	2	11.76	6	35.29	4	23.53

Overall, most participants reported finding all services strongly interesting except speechreading training and FM systems which the majority of participants found a little interesting.

### Audiologists’ views on challenges in aural rehabilitation services

**FIGURE 3:**
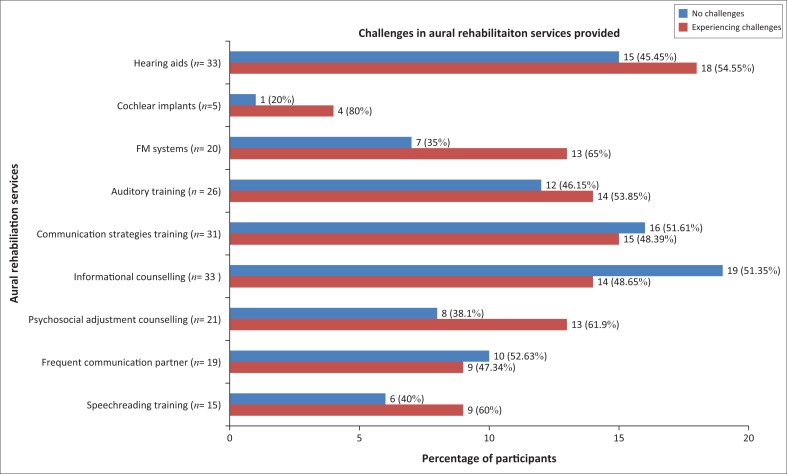
Challenges in aural rehabilitation services currently provided.

Challenges were experienced with all services as there were more participants that reported experiencing challenges with most services than those that did not (Figure 3).

Significantly more (*p* = 0.023) participants experienced challenges with auditory training than with speechreading training. A significant difference (*p* = 0.006) between communication strategies training and speechreading was indicated, with more participants experiencing challenges with the former. This was expected as more participants reported challenges with the service that was provided by markedly more participants.

Twenty-one participants specified challenges experienced. Of these, six (28.57%) reported client compliance and five (23.8%) reported lack of skills, knowledge or training in the field of aural rehabilitation. Four participants (19.04%) reported a language barrier, two (9.52%) reported unrealistic expectations from their clients, while another two (9.52%) reported individual differences amongst their clients. Another two (9.52%) participants reported the cost to their clients as the main challenge. Other challenges reported included affordability, limited time, finances and resources, language barrier, limited skills and knowledge in the field of aural rehabilitation, lack of motivation and poor compliance from clients. Training for service provision as a challenge was further explored. Responses are seen in Table 2.

**TABLE 2:** Undergraduate training for specific AR services.

AR service	Was never trained		Poorly trained		Sufficiently trained		Well trained		Very well trained	
	*n*	%	*n*	%	*n*	%	*n*	%	*n*	%
Hearing aids (*n* = 40)	3	7.5	7	17.5	15	37.5	9	22.5	6	15
Cochlear implants (*n* = 37)	22	59.5	13	35.1	1	2.7	0	-	1	2.7
FM systems (*n* = 37)	12	32.4	18	48.6	5	13.5	1	2.7	1	2.7
Auditory training (*n* = 38)	4	10.5	14	36.8	10	26.3	8	21.1	2	5.3
Communication strategies training (*n* = 37)	0	-	11	29.7	16	43.2	8	21.6	2	5.4
Informational counselling (*n* = 40)	1	2.5	8	20	15	37.5	12	30	4	10
Psychosocial adjustment counselling (*n* = 38)	1	2.6	13	34.2	12	31.6	10	26.3	2	5.3
Frequent communication partner training (*n* = 37)	4	10.8	12	32.4	9	24.3	10	27	2	5.4
Speechreading (*n* = 37)	7	18.9	15	40.5	7	18.9	5	13.5	3	8.1

Overall, most participants felt poorly trained for most aural rehabilitation services. The majority reported being sufficiently trained to provide hearing aids, communication strategies training and informational counselling. However, the majority also reported poor undergraduate training for the provision of FM systems, auditory training, psychosocial adjustment counselling, frequent communication partner training and speechreading training. Twenty-two (59.5%) of the 37 participants that responded reported that they were never trained for the provision of cochlear implants at all.

### Relationship between service provision, interest and challenges in aural rehabilitation

A relationship between service provision and interest as well as service provision and challenges experienced were explored to determine if, and the extent to which, these influence service provision (Table 3).

**TABLE 3:** Relationship of service provision, interest and challenges in each AR service.

AR service provided	Interest versus service provision (*p*-value)	Challenges versus service provision (*p*-value)	Interest versus challenges (*p*-value)
Hearing aids	0.020*	0.000*	0.026*
Cochlear implants	0.000*	0.000*	0.000*
FM systems	0.000*	0.000*	0.001*
Auditory training	0.335	0.050*	0.007*
Communication strategies training	0.014*	0.000*	0.152
Informational counselling	0.100	0.001*	0.150
Psychosocial adjustment counselling	0.004*	0.000*	0.012*
Frequent communication partner training	0.050*	0.013*	0.010*
Speechreading training	0.011*	0.001*	0.000*

* *p* ≤ 0.05

Overall, there was a significant difference between service provision, interest and challenges experienced by the participants with most services.

No statistically significant difference was found between interest and service provision for auditory training and informational counselling, possibly indicating an influential relationship between these variables for each service. Thus, it is likely there was high numbers of audiologists providing these services owing to their interest in them.

Similarly, there was no significant difference between interest and challenges for informational counselling and communication strategies training, indicating a possibility of interest being influenced by challenges or vice versa. Thus, it was likely that challenges had an impact on how interesting each service is found to be.

## Discussion

The study results provided insight into aural rehabilitation even though only 45 participants responded which limited the generalisability of the study. A possible reason for a poor response rate included the questionnaire being accessible only online meaning that some audiologists had to use their own internet and computers without any incentive. It is also likely that the topic was only of interest to those that responded. However, results provided a starting point from which more research can build on.

### Aural rehabilitation service provision

There was unequal service provision overall with hearing aids, communication strategies training and informational counselling being the most commonly provided services.

Amongst sensory management devices, intervention through hearing aids is, and has been, the most provided service traditionally, even in South Africa (Carmen, 2003; Hull, 2013; Naidoo, 2006; Ross, 2001). Cochlear implants were the least provided sensory management service possibly because of the need for additional training for South African audiologists to practice in this area. The reason for most audiologists not obtaining such training is to be investigated further. Another South African study (Naidoo, 2006) that investigated audiology service provision in the country also indicated that 91.67% of audiologists reported not providing cochlear implant services since nearly a decade ago. Thus, there could be very limited progress and expansion of cochlear implant services in the country as there are still very limited providers of this service. Likewise, FM systems are still provided by only a few audiologists. Although specific reasons for limited provision of FM systems need to be explored, it is suspected that it could be linked to limited provision of auditory processing assessments conducted prior to recommending an FM system (Naidoo, 2006). Many medical aids in South Africa do not cover the cost of FM systems, further limiting accessibility even in private practice. In comparison, provision of FM systems was and could still be at a much higher rate in the United States as the study conducted by Kelly and Prendergast (2002) indicated that 84% of participants provided assistive listening devices which include FM systems in the United States.

Most participants had reported being sufficiently trained for communication strategies training, which is consistent with it being the most provided amongst all communication training intervention services.

Auditory training seems to be provided by fewer audiologists over time in South Africa as only 44.2% of the participants in the current study in comparison with the 90% of government audiologists and 70% of private audiologists in the study by Naidoo (2006) provided it. Reasons for such a trend are not clear. However, the differences of sample sizes between the two studies could also have contributed to the findings. It is also likely that improved hearing aid technology could make auditory training less relevant as some participants in the study reported.

Speechreading training seems to be consistently amongst the least provided services, both abroad and in South Africa (Bally & Bakke, 2007; Naidoo, 2006; Prendergast & Kelly, 2002; Schow *et al*., 1993). Wemmer (2007) reported that many (26.28%) audiologists are only somewhat clinically prepared to provide speechreading services. In the current study, the majority (40.5%) reported poor undergraduate training and time constraints as reasons for not providing speechreading training. Again, the relevance of speechreading in the current times with better hearing aid technology is also in question. In practice, speechreading training takes much more time and effort in comparison with other communication training services, which could also explain its limited provision locally as audiologists are very limited.

The provision of frequent communication partner training is still not optimal as more than 50% of the audiologists do not provide it, possibly because of limited undergraduate training. Likewise, limited training has possibly led to limited psychosocial adjustment counselling in comparison to informational counselling which is provided by significantly more audiologists. This is consistent with literature which indicated that informational counselling is the most provided counselling compared with other types of counselling (Millington, 2000; Prendergast & Kelly, 2002; Ratanjee, 2014).

With regard to other technologies used in aural rehabilitation, tele-audiology and computer-aided aural rehabilitation programmes seem to be used to a limited extent locally as suggested by the current study results. It could be that knowledge and resources necessary to use such a technology are limited in the South African context. Only the LACE programme was reportedly used by less than 30% of participants that responded. None of the participants in the current study used the CATS, CASPERsent and CAST programmes. Most of these programmes were developed outside Africa and as such may not be seen as contextually relevant (Khoza, Ramma, Mphosho & Moroka, 2008; Pascoe & Norman, 2011). However, their use would be better than not, until locally relevant material is available.

Most participants did not use tele-audiology because they did not see a need for it and lacked knowledge or the necessary skills to use it. Other factors that influence the choice of material or technology to use when providing aural rehabilitation services are not known yet, especially in the South African context.

### Interest and challenges in aural rehabilitation

A strong interest in aural rehabilitation was generally reported. Thus, it is highly likely that most audiologists are interested in aural rehabilitation. However, this interest has not directly translated into increased service provision across all services, possibly because of limited undergraduate training for most services and other factors posing a challenge.

None of the aural rehabilitation services are without challenges with linguistic barriers highlighted. Language barrier and cultural diversity in South Africa are examples of a need to develop contextually relevant resources as research has indicated that the accuracy of a clinical tool could be maintained when made linguistically and culturally relevant (Pascoe *et al*., 2010; Rogers *et al*., 2011). In addition, challenges such as limited training, time, resource and financial constraints reported are expected to have negative effects on aural rehabilitation service provision. However, challenges and interest in isolation do not themselves have a significant effect in the provision of aural rehabilitation. Thus, a group of factors significantly affect service provision, and these factors need to be investigated further. The overall findings of this study indicated that although the audiology as a profession has come a long way in the development and provision of particular audiology services, there is still a great need for further development, particularly in training for, developing and providing aural rehabilitation services suitable for the South African context.

## Conclusion

Aural rehabilitation services are not provided optimally locally, with some services provided much less than others. This has implications for holistic and comprehensive aural rehabilitation. Challenges are likely experienced by most audiologists but challenges in isolation may not be sufficient to limit service provision. Further, local audiologists most likely have not started using technological developments to their full potential to improve efficiency of service provision. High interest in aural rehabilitation with limited service provision indicates a need to review various aspects of each service and its relevance to the current times and needs of clients with hearing loss. The study highlights a strong need for improvement in training and service provision for aural rehabilitation to fully benefit adults with hearing loss in South Africa.

### Limitations

The study had limited participants and was conducted via a survey where not all participants may have had easy access to the electronic questionnaire, limiting the generalisability of results. Limited numbers of participants limited the use of inferential statistics where inferences had to be made with caution.
